# Evolutionary Theory and Machine Learning: New Inroads Into Sex‐specific AD Susceptibility Genes and Pathways Significantly Improve Risk Prediction

**DOI:** 10.1002/alz.087889

**Published:** 2025-01-03

**Authors:** Ismael Al‐Ramahi, Jenn Asmussen, Juan Botas, Panagiotis Katsonis, Yashwanth Lagiesetty, Kwanghyuk Danny Lee, Fatemeh Nazem, Maryam Samieinasab, Chen Wang, Kevin Wilhelm, Olivier Lichtarge

**Affiliations:** ^1^ Jan and Dan Duncan Neurological Research Institute, Texas Children’s Hospital, Houston, TX USA; ^2^ Baylor College of Medicine, Houston, TX USA; ^3^ University of Texas Health, McGovern Medical School, Houston, TX USA

## Abstract

**Background:**

Alzheimer’s Disease (AD) incidence is almost double in female than male, suggesting sex‐specific AD risk genes remain unknown.

**Method:**

We designed a statistical physics approach that exploits freely available but massive evolutionary and phylogenetic coupling data on sequence variation and speciation. These couplings lead to quantifiable values for the selection pressure exerted on the genes within a population. We may then compare a gene’s influence in sequenced cases vs controls cohorts and test the hypothesis that significant deviations identify genes linked to disease risk.

**Result:**

In 4768 AD cases and 4689 healthy controls (HC), we discovered 122 genes under greater selection pressure (q < 0.01). These genes overlapped (p = 3.10^‐5^) and interacted (z = 7.16) with AD GWAS genes. They also interacted mutually (n = 57, p = 0.0019) and with AD‐related processes (p = 1.0^‐16^). More than 50% of the genes exhibited dysregulation in AD brains in snRNAseq analysis, suggesting participation in pathogenic or protective processes in AD. Furthermore, expression of these genes correlated with increased or decreased deposition of plaques and tangles in patient brains. Moreover, the candidates are enriched in modifiers of neurodegeneration in *Drosophila*: knockdown or overexpression of 64 genes ameliorated or worsened age‐dependent neuronal dysfunction (p<0.05). Robustness to down‐sampling allowed to analyze smaller, sex‐separated cohorts. We identified 82 genes in males and 69 genes in females (15 genes overlapped, p < 10^‐53^), indicating shared as well as sex‐specific AD mechanisms. The male and the female genes overlapped (p = 6.5^‐6^ and 1.5^‐4^) and interacted (z = 5.63 and 6.18) with the AD genes. Remarkably, using these gene sets as features for predicting AD risk in separate training and testing cohorts successfully differentiated between AD cases and controls with very high accuracy, even when blinded to APOE genotype. Notably, we predicted the risk with AUCs of 0.83 with APOE, 0.83, and 0.82 in combined, males, and females, respectively.

**Conclusion:**

A new statistical physics approach discovered male and female AD genes, predicting AD risk with very high accuracy. These results identify further genetic differences leading to AD in males and females, and show the power of quantitative phylogenetics to probe complex human diseases.

